# Cuticular Drusen and the Risk of Progression to Late Age-Related Macular Degeneration: A MACUSTAR Study Report

**DOI:** 10.1016/j.xops.2026.101375

**Published:** 2026-08-18

**Authors:** Lourdes Vidal-Oliver, Jan Terheyden, Charlotte Behning, Vanessa Basten, Maria Kornau, Lukas Schlößer, Ulrich F.O. Luhmann, Klaus Peter Moll, Nadia Zakaria, Sergio Leal, Matthias Schmid, Steffen Schmitz Valckenberg, Frank Holz, Robert P. Finger, Marlene Saßmannshausen, MACUSTAR consortium

**Affiliations:** 1 Vitreoretinal Unit, Fundación Oftalmología Médica Comunitat Valenciana, Valencia, Spain; 2 Department of Ophthalmology, Medical Faculty Mannheim, University of Heidelberg, Mannheim, Germany; 3 Department of Ophthalmology, University Hospital Bonn, Bonn, Germany; 4 Department of Medical Biometry, Informatics and Epidemiology, Medical Faculty, University of Bonn, Bonn, Germany; 5 GRADE Reading Center Bonn, Bonn, Germany; 6 Roche Pharmaceutical Research and Early Development, Translational Medicine Ophthalmology, Roche Innovation Center Basel, Basel, Switzerland; 7 Novartis Institutes for Biomedical Research, Cambridge, Massachusetts; 8 Bayer Consumer Care AG, Basel, Switzerland; 9 John A. Moran Eye Center, Department of Ophthalmology & Visual Sciences, University of Utah, Salt Lake City, Utah

**Keywords:** Cuticular drusen, Intermediate age-related macular degeneration, Progression

## Abstract

**Purpose:**

To analyze the prevalence of cuticular drusen (CD) and their relationship with other structural biomarkers in the progression to late age-related macular degeneration (AMD) within the MACUSTAR study (ClinicalTrials.gov Identifier: NCT03349801).

**Design:**

Longitudinal European multicenter cohort study.

**Subjects:**

585 study participants at a mean age of 72.1 ± 7.0 years with intermediate age-related macular degeneration (iAMD) at baseline visit.

**Methods:**

Using multimodal imaging, study eyes with iAMD were graded at baseline visit for CD and other AMD-associated biomarkers, including retinal pigment epithelium-drusencomplex (RPEDC) volume, reticular pseudodrusen (RPD), hyper-reflective foci (HRF), and pigmentary abnormalities (PA). We analyzed the prevalence of structural biomarkers according to CD status. Multivariable time-discrete hazard models were used to assess the association of CD alongside RPEDC volume, RPD, HRF, and PA on the progression to late-stage AMD within a 4-year follow-up period.

**Main Outcome Measures:**

Hazard ratio (HR) of progression from intermediate to late-stage AMD.

**Results:**

At baseline, 15.9% of iAMD eyes (93/585 participants; age 71.0 ± 7.2 years) presented with CD, with the following phenotype distribution: type 1, 45.2%; type 2, 32.2%; and type 3, 22.6%. RPD was less prevalent in eyes with CD (11.8%) compared to eyes without (29.9%), whereas other structural biomarkers were comparably prevalent between individuals with and without CD. Progression to late AMD occurred in 6% of eyes with CD (*n* = 6) and 16.0% without CD (*n* = 78). CD alone did not significantly modify the risk of AMD progression (HR = 0.49; 95% confidence interval [CI], 0.21‑1.12; *P* = 0.09). However, coexisting CD and RPD showed the highest risk (HR, 5.9; 95% CI, 1.1–31.5; *P* < 0.04), exceeding RPD alone (HR, 3.5; 95% CI, 2.3–5.5; *P* < 0.0001) or RPEDC volume (HR, 1.3; 95% CI, 1.1–1.5; *P* = 0.01). The presence of HRF and PA on top of CD did not significantly impact the risk of progression beyond the presence of HRF or PA alone. Over time, CD showed coalescence (37.6%), resorption (19.4%), and pigmentary changes (28.0%).

**Conclusions:**

In iAMD, CD alone does not significantly modify the risk of progression to late AMD in the absence of RPD. However, when CD and RPD coexist, the risk of progression to late AMD is increased. Further investigations, including genetic analyses, are needed to better understand the mechanisms underlying this association.

**Financial Disclosure(s):**

Proprietary or commercial disclosure may be found in the Footnotes and Disclosures at the end of this article.

Late-stage age-related macular degeneration (AMD) represents a significant cause of vision loss worldwide.^1^^,^^2^ In the context of a large unmet need to prevent vision loss in AMD, the body of evidence on the key drivers of progression of early AMD stages, including early and intermediate, AMD is growing.^3^ A variety of imaging biomarkers can help stratify patients with early AMD stages, including drusen load, pigmentary abnormalities (PA), hyper-reflective foci (HRF), and drusen subtype.^4^, ^5^, ^6^, ^7^

Cuticular drusen (CD) were first described by Gass as multiple small, uniform, yellow deposits with a “starry sky” appearance on fluorescein angiography (FA) but are still poorly understood.^8^ Compared with conventional drusen, CD are more frequent in women, appear in younger individuals, can be found scattered around the fundus, and might be more influenced by genetic rather than environmental factors.^9^, ^10^, ^11^ Individually, CD are indistinguishable from druplets in histological samples, but they are more numerous, tend to aggregate, and have more clinical implications.^12^ To date, CD has rarely been studied in isolation from other macular deposits.^7^^,^^12^, ^13^, ^14^, ^15^, ^16^, ^17^, ^18^

Differentiating CD from other drusen subtypes could be clinically relevant, but published studies have presented conflicting evidence regarding their prognostic significance.^19^ The largest analysis published to date indicated that the presence of CD did not influence the risk of progression to late AMD or declining retinal sensitivity beyond soft drusen.^15^ Similarly, the Amish Eye Study reported that, although CD were highly prevalent among eyes with nonadvanced AMD, they were not independently associated with 2-year progression to late AMD.^7^ In contrast, recent data from an Asian neovascular AMD (nAMD) cohort associated CD with polypoidal choroidal vasculopathy, suggesting that the clinical expression of CD may vary across populations and may be influenced by coexisting features.^18^ Together, these findings suggest that the prognostic relevance of CD may depend less on the mere presence of CD and more on the broader phenotypic context in which CD occurs. The interaction effect between CD and other structural biomarkers has not yet been systematically investigated.

The current analysis was conducted within the MACUSTAR study, a large, multicenter European cohort with standardized multimodal imaging and longitudinal follow-up in intermediate age-related macular degeneration (iAMD).^3^ Although previous MACUSTAR reports described the baseline characteristics and prevalence of major structural biomarkers such as PA, reticular pseudodrusen (RPD), and HRF,^20^ CD had not been specifically analyzed. A dedicated characterization of CD within this cohort may therefore provide additional insight into the structural phenotyping of iAMD.

We have hypothesized that the conflicting literature results regarding progression risk in AMD patients with CD could be explained by different underlying phenotypes in individuals with CD that modify the risk of progression in different directions. We have, thus, analyzed the interaction effects between CD and other structural biomarkers on the risk of AMD progression and the longitudinal progression of CD over time in the large, multicenter cohort of iAMD in the context of the MACUSTAR study.^21^

## Methods

Our report presents an analysis of CD in eyes with iAMD in the MACUSTAR study cohort, with a particular focus on the longitudinal assessment of up to 4 years of follow-up. For the current analysis, all iAMD study eyes of the longitudinal part of MACUSTAR were included, comprising all iAMD patients from the cross-sectional part (n = 168), and additional 417 newly recruited iAMD patients, resulting in a total of 585 iAMD study eyes.

The primary objective is to examine the influence of CD presence alongside other structural AMD risk biomarkers at baseline on the progression from iAMD to late AMD. As secondary outcomes, we investigated the association between CD and other structural biomarkers and other demographic factors that may influence the presence of CD, such as age, sex, and country of recruitment. We also assessed CD phenotypes and their longitudinal changes over time. Functional measures, including best-corrected visual acuity (BCVA) and low-luminance visual acuity (LLVA), were additionally analyzed according to the presence of CD.

### The MACUSTAR Study

MACUSTAR is a European multicenter, low-interventional clinical study designed to establish new regulatory acceptable clinical endpoints for iAMD (ClinicalTrials.gov Identifier: NCT03349801). The detailed study design, inclusion criteria, and the detailed protocol can be found elsewhere.^3^^,^^21^ Briefly, the study consists of a cross-sectional and a longitudinal part. Patients aged 55 to 85 years at baseline with a Snellen BCVA better than 20/40 in the iAMD cohort were included. The cross-sectional part included participants with different stages of AMD (early, intermediate, and late), and age-matched controls with the primary objective of technical evaluation of functional and structural candidate outcomes. The longitudinal part included study participants and study eyes from the early and intermediate AMD cross-sectional study cohorts as well as additional enrolled iAMD participants with a follow-up time of 6 years (6-year data collection ongoing until 2026). In the fellow eyes of the iAMD study cohort, presence of extrafoveal geographic atrophy (GA) up to 1.25 mm^2^ was permitted in the cross-sectional part, whereas any AMD stage was eligible within the longitudinal study cohort.

The aim of the longitudinal study part is to evaluate the prognostic value of functional, structural, and patient-reported outcomes for progression to late-stage AMD. In this report, we included data from 4 years of follow-up time. The Beckman classification was used to classify AMD stages.^22^ Early AMD was defined as having medium-sized drusen (63‑125 μm), whereas iAMD required large drusen (>125 μm) and/or PA. The same AMD stage was required in both eyes of the same patient, with the eye with the best BCVA selected as the study eye. As an exception, an area of extrafoveal GA <1.25 mm^2^ was allowed in the fellow eye of the iAMD cohort. Late AMD was defined as either GA or exudative AMD.^21^

The study protocol was approved by the institutional review boards of all participating centers. The study was conducted in accordance with the Declaration of Helsinki, and all patients signed an informed consent form prior to participation.

### Imaging Protocol

At each visit, a detailed and standardized multimodal imaging protocol was performed by certified personnel at each study site. After pupil dilation, a Spectralis Heidelberg Retina Angiograph-OCT device (Heidelberg Engineering) was used to obtain near-infrared, fundus autofluorescence (FAF), and spectral-domain OCT (SD-OCT). For near-infrared and FAF, a 30 × 30° area centered on the fovea was imaged with an automatic real-time mode of *≥*30 frames. For OCT, 2 different scan patterns were used: a standard SD-OCT raster scan with a size of 20 × 20°, consisting of 25 B-scans with an interscan distance of 240 μm, and a dense scan in the enhanced depth imaging mode with a size of 30 × 25°, consisting of 241 B-scans separated by 30 μm. Color fundus photography (CFP) was conducted on all study participants at each visit. Fluorescein angiography was performed on an as-needed basis in cases of suspected conversion to late AMD according to physician criteria.

All images were transferred to the central reading center (GRADE Reading Center, Bonn, Germany) via a secure server. Standardized grading of retinal imaging data was performed by one junior reader, followed by one senior grading review according to a standardized and predefined grading procedure manual. Grading by readers comprised AMD staging as well as the presence of various retinal imaging biomarkers like HRF, PA, vitelliform material, refractile deposits, RPD, retinal pigment epithelium (RPE) detachment, incomplete or complete RPE and outer retinal atrophy, and CD. Retinal pigmentepithelium-drusen complex volumes were analyzed using a deep learning-based approach. Retinal pigment epithelium-drusen complex volumes were classified as abnormal when the corresponding retinal pigment epithelium-drusen complex (RPEDC) thickness was ≥3 standard deviations (SDs) above the control group mean. This was quantified across the full OCT volume (241 B-scans). Detailed information on the multimodal retinal imaging-based definition of structural biomarkers and on the grading process was described in previous reports.^23^^,^^24^

### Grading of Cuticular Drusen

For the grading of the presence of CD, a multimodal retinal imaging approach was performed, including the assessment of the 241 B-scans, SD-OCT, FAF, and CFP imaging. The reliability of our methodology was subsequently validated by comparing our masked grading strategy, which used only CFP, SD-OCT, and FAF, with the reference standard of FA in the subgroup of cases where it was available.

In SD-OCT, CD were identified as RPE elevations and classified according to previously described criteria by Balaratnasingam et al^12^ into 3 subtypes: shallow RPE elevations with indistinct internal contents (type 1); triangular, saw-tooth RPE elevations with hyporeflective internal contents and RPE thinning at the apex (type 2) (Fig 1); and broad, mound-shaped hyporeflective RPE elevations (type 3). Eyes were considered CD-positive when ≥5 CDs were identified within the macular raster OCT scan (30 × 25°, 241 B-scans, spacing 30 μm).

**Figure 1:**
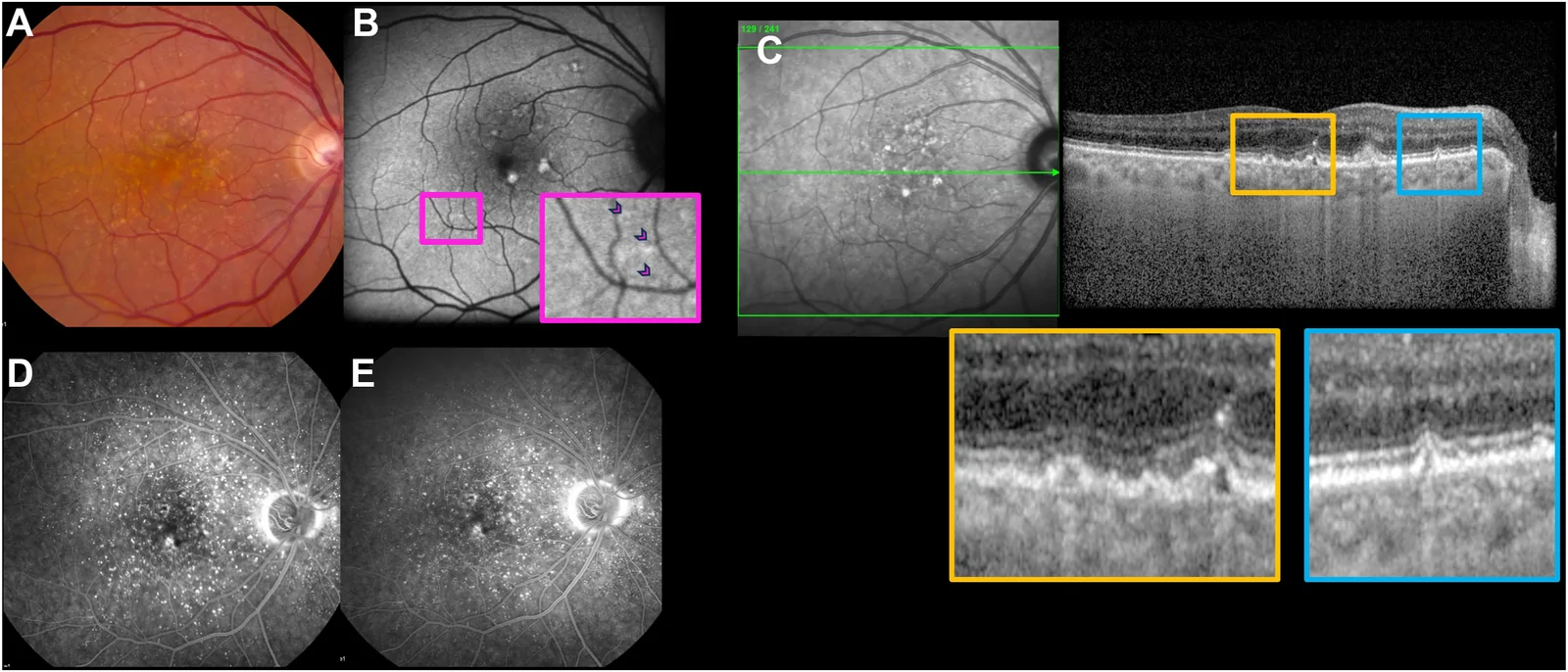
Multimodal retinal imaging approach for the assessment of cuticular drusen (CD) presence in color fundus photography (**A**), fundus autofluorescence (**B**), infrared, and OCT imaging (**C**; first row, from left to right). Note the typical CD appearance as hypoautofluorescent spots with a hyperautofluorescent rim in the fundus autofluorescence imaging (**B**, highlighted by pink arrows in the enlarged image section). In SD-OCT imaging (**C**) CD appear as retinal pigment epithelium (RPE) elevation with a triangular, saw-tooth configuration, with hyporeflective internal content and RPE thinning at the apex, as highlighted in the yellow- and blue‑bordered image section. Additional within the study course available fluorescein angiography (second row, **D** and **E** after 1-minute and 3-minute early phase) confirmed CD presence with the presentation of the characteristic “stars in the sky” fundus pattern. SD-OCT = spectral-domain OCT.

In CFP, CD were defined as multiple, small, clustered, roundish-yellow lesions (Fig 1A). Nevertheless, our definition did not exclude the possibility of larger deposits that have formed because of the coalescence of CD or midperipheral colloidal drusen. The distribution of CD may be either concentrated in the macula or scattered throughout the fundus, including the peripapillary area.^16^ On FAF imaging, typical CD appear as hypoautofluorescent spots with a hyperautofluorescent rim (Fig 1B).

Additionally, longitudinal changes in CD over time were also assessed, including CD resorption or disappearance, appearance of pigmentary changes, and CD coalescence.

Detailed protocols for functional assessments within the MACUSTAR study, including BCVA and LLVA, have been described in previous reports.^3^^,^^25^

### Statistical Analysis

All statistical analyses were performed using the R software environment (R Foundation, Vienna, Austria, version 4.0.2). Baseline characteristics were expressed as frequencies and means with SDs. The pairwise association between CD and other structural biomarkers in the iAMD group was calculated using Pearson's χ^2^ test with Yates' continuity correction. A multivariable time-discrete subdistribution hazard model with a complementary log-log link function was employed to investigate the progression to late AMD.^26^ Death and intraocular conditions that would confound the study outcomes were incorporated as a composite competing event. Time since baseline was modeled as a penalized spline (with second-order difference penalty and 5 equidistant interior knots), allowing for nonlinear effects of time. Event occurrence was assessed at discrete follow-up visits (interval censoring at t = 2, 4, 5, 6, …, 11, corresponding to 0, 6, 12, 18, …, 48 months of follow-up, respectively). After event occurrence, individuals were removed from the risk set. Subdistribution weights were applied to account for competing risks, thereby implementing a Fine–Gray type subdistribution hazard approach in discrete time.^27^ The presence of CD, age, and RPEDC volume at baseline were identified as the independent variables, along with the presence of other structural biomarkers (including HRF, RPD, and PA). With 84 observed progression events, the resulting events-per-variable ratio was well above the commonly recommended threshold of 10. Furthermore, models additionally including an interaction effect of CD and the aforementioned biomarkers were fitted. RPEDC volumes were z-scaled prior to analysis (centered to mean = 0 and scaled to SD = 1). As a result, values are unitless and represent standardized deviations from the cohort mean.

We included only RPEDC volume, HRF, RPD, and PA as independent variables because these are the imaging features most associated with disease progression according to previous studies.^4^^,^^28^, ^29^, ^30^ Study participants without documented disease progression at their last available study visit, including those lost to follow-up, were right-censored at the time of their last assessment.

Intergrader agreement for CD grading was evaluated using Gwet’s AC1 coefficient.^31^

Statistical significance was defined at *P* values <0.05.

## Results

### Baseline Characteristics

A total of 585 study eyes from 585 participants with iAMD (mean age: 72 ± 7 [SD]; median, 72 [range, 55‑88] years at baseline visit; 66.5% women) were included. Baseline demographics and the prevalence of key structural biomarkers—including PA (49.1%), RPD (27.0%), and HRF (47.5%)—have been reported previously in the cross-sectional MACUSTAR baseline analysis.^20^ Within the current analyzed cohort, the overall prevalence of CD was 15.9%, corresponding to 93 of 585 iAMD participants. The mean age of iAMD study participants in presence and absence of CD was overall comparable, with a mean of 71.0 ± 7.2 (SD) years versus 72.3 ± 7.0 (SD) years.

Among eyes with CD, the most common OCT-based phenotype was shallow RPE elevations (type 1), observed in 42 eyes (45.2%), followed by the saw-tooth pattern (type 2) in 30 eyes (32.2%) and broad hyporeflective (type 3) in 21 eyes (22.6%).

Intergrader agreement for CD between junior and senior readers was high (Gwet’s AC1, 0.72–0.903), indicating good to very good agreement. Intergrader agreement for other structural biomarkers was also good, with Cohen’s Kappa values of 0-8-0.85 for PA, 0.6-0.42 for HRF, and 0.75 for RPD.

### Prevalences of Structural Biomarkers in Cuticular Drusen Study Eyes

Comparing prevalence rates between iAMD study eyes with and without CD, the presence of RPD was significantly lower in eyes with CD (11.8% with CD vs. 29.9% without CD; *P* < 0.0001). Prevalence rates of PA, HRF vitelliform lesions, refractile deposits, and PED lesions were comparable between both study eye groups, as were the z-scored RPEDC volume values.

Detailed cohort characteristics and prevalence rates of structural parameters in study eyes in presence and absence of CD are given in Table 1.

**Table 1:** Patient Characteristics and Structural Parameters of the iAMD Cohort Stratified by the Presence of Cuticular Drusen

	With CD (n = 93)	Without CD (n = 490)
Sex (female) [N, %]	72 (77.4%)	317 (64.4%)
Age (yrs) [mean (SD), range]	71.0 (7.23) [56, 84]	72.3 (6.97) [55, 88]
BCVA (LogMAR) [mean, SD]	0.04 (0.11)	0.03 (0.11)
Presence of PA (N, %)	41 (44.1%)	246 (50.0%)
Presence of vitelliform material (N, %)	1 (1.1%)	20 (4.1%)
Presence of HRF (N, %)	42 (45.2%)	236 (48.0%)
Presence of refractile deposits (N, %)	9 (9.7%)	25 (5.1%)
Presence of RPD (N, %)	11 (11.8%)	147 (29.9%)
Presence of PED (N, %)	4 (4.3%)	25 (5.1%)
Presence of qCNV (N, %)	1 (1.1%)	7 (1.4%)
Presence of cystoid lesions (N, %)	1 (1.1%)	10 (2.0%)
Presence of GA in the fellow eye (N, %)	6 (6.5%)	28 (5.7%)
RPEDC volume (mean, SD)	–0.153 (0.63)	0.024 (1.04)

BCVA = best-corrected visual acuity; CD = cuticular drusen; GA = geographic atrophy; HRF = hyper-reflective foci; iAMD = intermediate age-related macular degeneration; LogMAR = logarithm of the minimum angle of resolution; PA = pigmentary abnormalities; PED = retinal pigment epithelium detachment; qCNV = quiescent macular neovascularization; RPD = reticular pseudodrusen; RPEDC = retinal pigment epithelium-drusen complex; SD = standard deviation.

RPEDC volume shown as z-scores (standardized).

### Functional Evaluation

Functional assessment demonstrated greater visual impairment in study eyes with RPD and combined CD and RPD compared to study eyes with CD alone. More pronounced differences were observed for LLVA than for BCVA testing. Detailed results of functional testing across subgroups (CD only, RPD only, CD + RPD, and neither CD nor RPD) at the baseline visit are provided in Table 2.

**Table 2:** Functional Testing

	CD Only (n = 82)	RPD Only (n = 147)	CD + RPD (n = 11)	CD-/RPD- (n = 343)	Overall (N = 583)
BCVA (logMAR) [mean, SD]	0.032 (0.108)	0.057 (0.101)	0.058 (0.080)	0.015 (0.104)	0.029 (0.105)
LLVA (logMAR) [mean, SD]	0.218 (0.180)	0.275 (0.156)	0.329 (0.184)	0.231 (0.146)	0.242 (0.156)

BCVA = best-corrected visual acuity; CD = cuticular drusen; LLVA = low-luminance visual acuity; logMAR = logarithm of the minimum angle of resolution; RPD = reticular pseudodrusen; SD = standard deviation.

Table 2 shows detailed results of functional testing across subgroups (CD only, RPD only, CD + RPD, and neither CD nor RPD) at the baseline visit.

### Regional Variety of Cuticular Drusen Prevalence

Distinguishing study patients according to the different participating European countries, there was a significantly higher prevalence of CD observed in Portugal (47% of eyes with CD) compared to central and northern European countries (e.g., 3% and 6% in Denmark) and the United Kingdom. A detailed distribution of CD according to the participating European countries is given in Table 3. Table S1 (available at www.ophthalmologyscience.org) shows the baseline characteristics by country.

**Table 3:** Regional Variations in the Prevalence of Cuticular Drusen in Eyes with iAMD

Country	Eyes with CD (Study Eyes with CD/Totally Enrolled Study Eyes by Country, %)
Denmark	1/30, 3%
United Kingdom	4/70, 5.7%
France	6/62, 9.7%
The Netherlands	6/55, 10.9%
Italy	11/87, 12.6%
Germany	31/209, 14.8%
Portugal	34/72, 47.2%

CD = cuticular drusen; iAMD = intermediate age-related macular degeneration.

### Progression to Late Age-Related Macular Degeneration

Over the course of the 4-year follow-up period, 126 of 585 iAMD patients have been lost to follow-up. 84 study eyes progressed from intermediate to late AMD (38 = GA and 46 = nAMD). Of those progressing to late AMD, 6 eyes had CD (representing 6.5% of the total number of study eyes with CD) and 78 without CD (16% of the study eyes without CD). Progression subtype and descriptive time-to-event information stratified by the presence of CD and RPD are summarized in Table 4.

**Table 4:** Progression to Late AMD Subtype and Descriptive Time-to-Event by Subgroup (CD Only, RPD Only, and CD + RPD)

Subgroup	GA		nAMD	
	N, Median Time to Event (yrs)	%	N, Median Time to Event (yrs)	%
CD only (n = 82)	1 (1.0)	1.2%	2 (2.75)	2.4%
RPD only (n = 147)	23 (2.0)	15.6%	17 (2.0)	11.6%
CD + RPD (n = 11)	1 (1.0)	9%	2 (1.5)	18.2%

AMD = age-related macular degeneration; CD = cuticular drusen; GA = geographic atrophy; nAMD = neovascular age-related macular degeneration; RPD = reticular pseudodrusen.

The presence of CD alone did not show a significant effect on progression to late AMD (hazard ratio [HR], 0.49; 95% confidence intervl [CI], 0.21‑1.12; *P* = 0.0908). Cumulative incidences of disease progression in dependence of structural biomarkers are illustrated in Table 5, Model 1.

**Table 5:** Multivariable Time-Discrete Hazard Models with the Progression to Late AMD (Atrophic and/or Exudative) as the Dependent Variable

	Model 1			Model 2		
	HR	95% CI	*P* Value	HR	95% CI	*P* Value
CD	0.49	0.21–1.12	0.089	0.50	0.22–1.16	0.108
RPD	3.52	2.25–5.50	**<0.001**	3.15	1.98–5.0	**<0.001**
PA	3.17	1.91–5.26	**<0.001**	2.93	1.74–4.95	**<0.001**
HRF	6.73	3.72–12.18	**<0.001**	6.47	3.48–12.03	**<0.001**
RPD × CD				5.92	1.11–31.51	**0.037**
PA × CD				1.86	0.21–16.95	0.581
HRF × CD				1.04	0.11–9.69	0.974

AMD = age-related macular degeneration; CI = confidence interval; CD = cuticular drusen; HR = hazard ratio; HRF = hyper-reflective foci; PA = pigmentary abnormalities; RPD = reticular pseudodrusen; RPEDC = retinal pigment epithelium-drusen complex.

Model 1 adjusted for age, and model 2 adjusted for age, RPEDC volume, and the presence of cuticular drusen, with interaction terms between each structural biomarker and cuticular drusen. Coefficient estimates for the intercept and age at baseline are not shown.

Significant *P* values are given in bold.

In the adjusted model for age, RPEDC volume, and the presence of CD (Table 5, Model 2), the presence of HRF was the structural biomarker most strongly associated with progression to late AMD. In this model, the likelihood of progression at any visit was approximately 7 times higher for those with HRF than for those without (HR = 6.7; 95% CI, 3.7–12.2; *P* < 0.0001). Similarly, eyes with PA were 3 times more likely to progress than those without PA (HR = 3.2; 95% CI, 1.9–5.3; *P* < 0.001). These findings remained consistent when the presence of CD was not included in the model (detailed results provided in Table 5).

No significant association was found for the interaction effect of HRF with CD (HR, 1.0; 95% CI, 0.11–9.7; *P* = 0.97) and PA with CD (HR, 1.9; 95% CI, 0.2–17.0; *P* = 0.58) for the risk of disease progression.

The coexistence of RPD and CD, although rare, had the highest cumulative incidence. Reticular pseudodrusen and CD occurred together in only 11 patients, of which 3 progressed to late AMD (2 to exudative AMD and 1 to GA; median time to progression, 1.5 years for nAMD and 1 year for GA) and the remaining were censored after 3 years of follow-up.

The model showed that the simultaneous presence of the 2 deposits was associated with a 2-fold increase in the risk of progression, adjusted for age and RPEDC volume (HR = 5.9; 95% CI, 1.1–31.5; *P* = 0.037), compared to eyes with RPD alone (HR, 3.2; 95% CI, 2.0–5.0; *P* < 0.001). Eyes without RPD but with CD presence demonstrated the lowest cumulative incidence (Fig 2).

**Figure 2:**
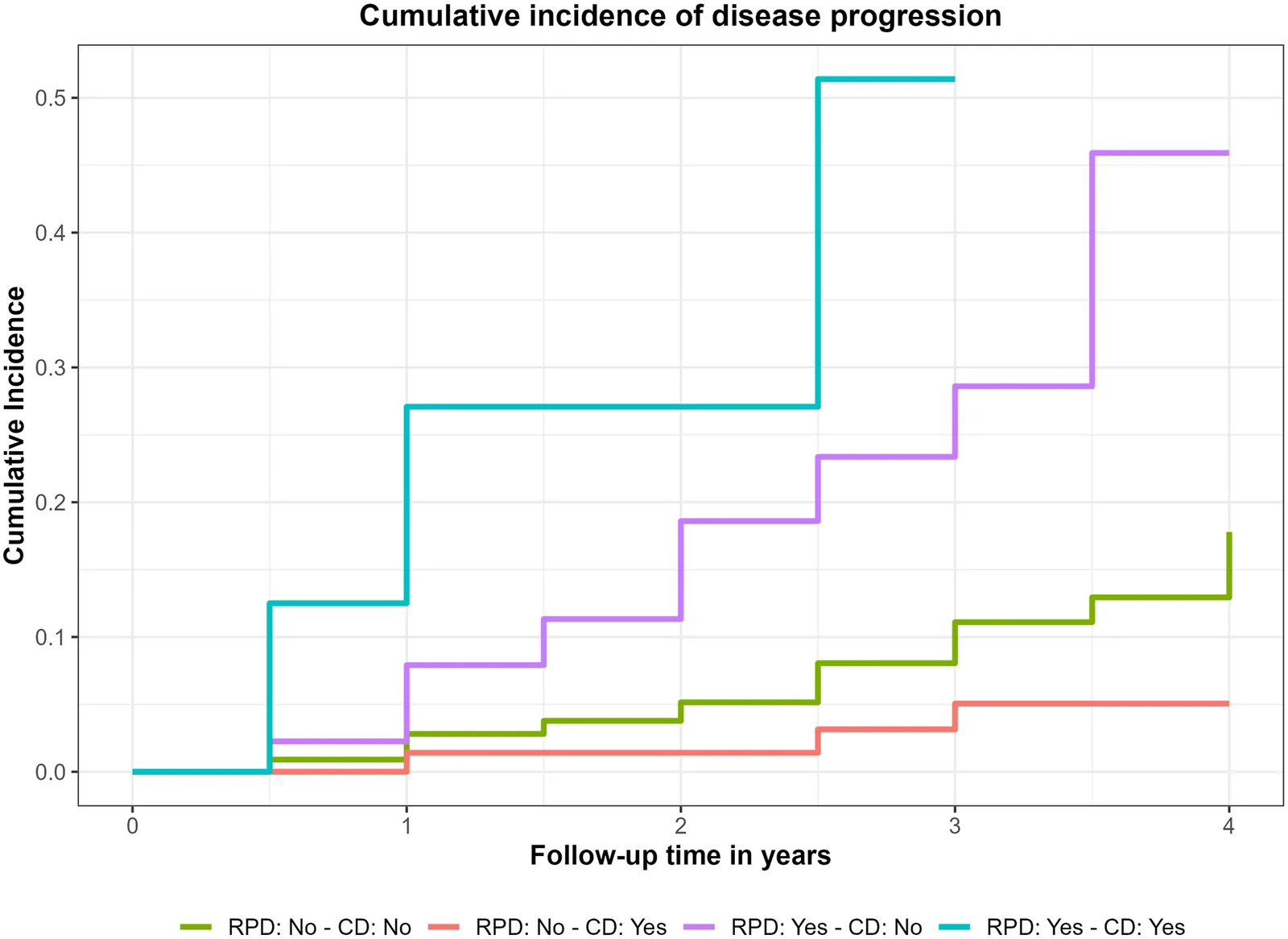
Graphical illustration of the cumulative incidence of disease progression in intermediate AMD study eyes stratified by the presence of reticular pseudodrusen (RPD) and cuticular drusen (CD). Note the overall increased cumulative incidence rate of disease progression in study eyes with both RPD and CD (in blue) compared to study eyes with only RPD present (purple). Overall, lowest incidence of disease progression was detected in study eyes with sole CD presence. AMD = age-related macular degeneration.

### Longitudinal Evolution of Cuticular Drusen

At 4 years of follow-up, among all eyes with CD (n = 93), 35 (37.6%) showed coalescence, 18 (19.4%) resorption, and 26 (28%) RPE changes. In eyes with coexisting CD and RPD (n = 11), CD phenotype and longitudinal evolution were further characterized according to baseline OCT appearance (Table 6).

**Table 6:** Baseline OCT Phenotype Distribution and 4-Year Longitudinal Evolution of Cuticular Drusen in Eyes with Coexisting CD and Reticular Pseudodrusen

Baseline CD Phenotype	Four-Year Longitudinal Evolution			Total (N, %)
	Coalescence (n)	Resorption (n)	RPE Changes (n)	
Shallow RPE elevations (type 1)	1	2	0	6 (54.5%)
Saw-tooth pattern (type 2)	0	0	0	1 (9.1%)
Broad hyporeflective (type 3)	1	3	2	4 (36.4%)
Total (N, %)	2 (18.2%)	5 (45.5%)	2 (18.2%)	11 (100%)

CD = cuticular drusen; RPE = retinal pigment epithelium.

Baseline CD phenotype according to the classification by Balaratnasingam et al.^12^

Among the 3 eyes with both CD and RPD that progressed to advanced AMD, one with type 3 CD showed coalescence and pigmentary changes over time and progressed to nAMD, one with type 1 CD showed drusen resorption and progressed to nAMD, and one with type 3 CD showed drusen resorption and progressed to GA. These cases were recruited in Germany, the United Kingdom, and France, with one case from each country.

## Discussion

This study examined the prevalence of CD at baseline visit within the MACUSTAR study and its role as a modifying factor in AMD progression. We report a prevalence of CD of 16% in the iAMD study cohort, showing a geographic distribution, with lower prevalence in northern European countries. Cuticular drusen, whether occurring alone or in combination with HRF or PA, do not appear to increase the risk of progression to late AMD. However, when CD coexist with RPD, the risk of progression is significantly higher.

### Cuticular Drusen Phenotypes and Longitudinal Progression of Cuticular Drusen

Few studies to date have stratified CD into subtypes based on OCT appearance. In the original OCT-based classification, Balaratnasingam et al reported type 2 as the most frequent phenotype (49%), followed by type 1 (33%) and type 3 (18%).^12^ In an Asian cohort, Shin et al reported a similar distribution.^4^ In contrast, type 1 was the predominant phenotype in our cohort. These differences may reflect variations in study populations. Notably, our cohort of eyes with CD was older than those reported by Balaratnasingam et al and Shin et al (mean age 71 years vs. 57.9 and 66.6 years, respectively), whereas type 2 lesions may be more common in younger individuals.^12^^,^^14^ Conversely, a recent Asian nAMD cohort reported a higher prevalence of phenotype 3 (33.3%).^18^ This suggests that OCT phenotype distribution may be influenced by age, ethnicity, and disease stage, as type 3 lesions may arise through drusen coalescence over time. Further studies are needed to better understand their relative contributions.

Regarding the natural history of CD, Balaratnasingam et al reported coalescence in 70.8% of eyes, resorption in 58.3%, and RPE changes in 56.2% over a mean follow-up of 10.5 years.^12^ In comparison, our prospective cohort of iAMD eyes had a shorter follow-up (4 years) but demonstrated a similar pattern, with coalescence being the most frequent change, followed by resorption and RPE alterations. With longer follow-up, these rates would likely increase. Overall, our findings support that CD are dynamic lesions and may behave similarly to soft drusen, undergoing morphological changes over time.

### Cuticular Drusen as a Risk Factor of Late Age-Related Macular Degeneration

In our cohort, 6.5% of eyes with CD progressed to late AMD. This is notably lower than the progression rates reported in previous studies, such as 12.5% to macular neovascularization and 25% to GA by Balaratnasingam et al and 23.8% to late AMD by Sakurada et al.^12^^,^^16^ However, these studies focused on the natural history of CD and did not include eyes without CD for comparison or account for other risk-modifying factors. In contrast, our findings provide a more comprehensive assessment of CD’s role in AMD progression within a broader clinical context.

Our study assessed progression from intermediate to late AMD based on the presence of various imaging biomarkers, including RPD, PA, and HRF, in combination with CD. We found that CD alone, adjusted for RPEDC volume and age—or in combination with PA and HRF—were not associated with an increased risk of progression to late AMD. This is consistent with previous prospective studies, including the sham group of the Laser Intervention in Early Stages of Age-Related Macular Degeneration (LEAD) Study and the Amish Eye Study cohort, which likewise found no independent association between CD and AMD progression. Instead, other biomarkers, such as drusen volume, PA, incomplete RPE and outer retinal atrophy, and acquired vitelliform lesions, were described as stronger predictors.^7^^,^^15^

To further investigate the role of CD, we included interaction terms between CD and other known AMD risk biomarkers, such as RPD. Interestingly, we found that the combination of CD and RPD conferred the highest risk of progression to late AMD—an observation that has not been described in the previous literature. By adjusting for RPEDC volume in our model, we show that these findings are not simply explained by a higher overall drusen load. Given the limited number of eyes with both CD and RPD, these results should be interpreted as exploratory and hypothesis-generating.

The interpretation of this finding is further complicated by differences across study populations. For example, a recent Asian nAMD cohort reported a higher prevalence of coexisting CD and RPD than observed in our European iAMD cohort (33.3% vs. 11.9% of the total eyes with CD).^18^ Whether this reflects differences in ethnicity, genetic background, disease stage, or a combination of these factors remains unclear. Previous studies have identified specific genetic alterations in eyes with CD, particularly involving risk alleles in the complement factor H gene.^10^^,^^11^ Conversely, eyes with RPD have been associated with a higher AMD polygenic risk score and were more often linked to non-CFH genetic variants than eyes without RPD.^32^^,^^33^ Thus, in patients presenting both CD and RPD, coexisting genetic factors may contribute to more severe structural and functional retinal damage and need to be further investigated in large clinical trials.

Supporting this, Yoon et al reported that eyes with both CD and RPD exhibited thinner subfoveal choroidal thickness compared to those with RPD alone.^34^ In our cohort, eyes with coexisting RPD and CD showed worse visual function than eyes with CD alone, for both BCVA and LLVA testing, and the poorest LLVA among all groups (Table 2). However, functional differences were broadly comparable to those observed in eyes with RPD alone, suggesting that RPD may be a major contributor to functional impairment. Although additional functional endpoints were not assessed in the present study, it is plausible that eyes with both CD and RPD represent a more vulnerable subgroup. Previous studies have suggested that CD is primarily associated with cone degeneration, whereas RPD is linked to rod dysfunction.^35^ The coexistence of both may, therefore, contribute to a greater functional burden, a hypothesis that warrants further investigation.

### Geographic Heterogeneity of Cuticular Drusen

In our study, 16% of study eyes with iAMD were graded as having CD, which closely matches the prevalence reported by Manafi et al in nonatrophic areas (15.8%),^36^ but is notably lower than the 25% prevalence observed by Goh et al in participants from the LEAD study.^15^ Like our study, both used various combinations of multimodal imaging—excluding FA—and performed a standardized grading of structural biomarkers.

These discrepancies in CD prevalence may be influenced by geographic variation. The MACUSTAR study, which includes participants from 7 European countries, allowed us to compare CD prevalence under standardized conditions across different regions. Imaging was acquired using a harmonized protocol and graded centrally in a masked manner, minimizing site-related bias. We observed a significantly lower prevalence of CD in northern countries such as the United Kingdom and Denmark (3%–5.7%), compared to higher rates in more southern regions: 9.7%–14.8% in France, the Netherlands, northern Italy, and Germany and 47.2% in Portugal.

Baseline characteristics stratified by the country of recruitment are provided in Supplementary Table S1. Although differences in demographic, genetic, referral-related, and environmental factors such as latitude or sunlight exposure may contribute to the observed variation in CD prevalence, the present study was not specifically designed to assess determinants in geographic variability. Furthermore, no additional stratification by age, sex, or recruitment site was performed in the models, as the limited number of CD cases would limit the robustness of any resulting conclusions. Therefore, these observations should be considered exploratory and hypothesis-generating. Nevertheless, the lack of association between CD only and progression risk is consistent with findings from the Australian cohort of the LEAD study.^15^

### Detection of Cuticular Degeneration on Multimodal Imaging

Cuticular drusen are classically identified by a “starry sky” pattern on FA, which remains the gold standard for diagnosis.^8^ However, in the context of early iAMD—where exudative signs are absent—routine FA is not typically justified in clinical practice. As in previous studies, we therefore employed a multimodal imaging approach to detect CD, requiring concordant findings across ≥3 imaging modalities, most commonly OCT, CFP, and FAF.^9^^,^^15^^,^^37^ This approach reflects current clinical practice in AMD and was supported by centralized grading performed independently by certified readers following harmonized protocols. In addition, internal validation data from 19 eyes of the MACUSTAR cohort with available FA images within the study course demonstrated high concordance between multimodal-imaging grading and FA findings in a masked evaluation, with only one case misclassified as CD-negative in the absence of FA. Nevertheless, because FA was available only in a limited subset of eyes, some degree of verification bias cannot be excluded, as eyes undergoing FA may have had a higher drusen burden or suspected neovascular disease.

This study has several limitations. First, the relatively small number of eyes with CD that progressed to late AMD limited our ability to separately assess the risk for GA and macular neovascularization, and reduced statistical power to detect an overall effect of CD, but our analysis considered all relevant risk biomarkers and was exploratory in nature. Second, the limited number of eyes with coexisting CD and RPD may have reduced the precision of the interaction estimates. Potential misclassification of CD or RPD status (although grading was performed independently by 2 certified readers following a standardized protocol) could influence the observed association, and the findings should therefore be interpreted with caution. Third, in the iAMD group, eligibility required relatively preserved visual acuity in the study eye (BCVA ≥ 20/40). Accordingly, the cohort primarily represents patients with earlier-stage disease, and findings may not fully generalize to more advanced AMD. Fourth, the 4-year follow-up period may not capture later conversions to advanced AMD, and longer observation could reveal additional progression events. Despite these limitations, our study benefits from a large overall sample size drawn from a prospective, longitudinal clinical trial, conducted under a standardized protocol across multiple geographic regions within Europe. To our knowledge, this is the first study to explore the interaction between CD and RPD in AMD progression and to report regional variations in CD prevalence. A further strength is the adjustment for drusen volume in the progression models, suggesting that the observed effects are not merely due to a higher drusen load but instead reflect the specific impact of coexisting CD and RPD on disease progression.

### Conclusions

We found that the presence of CD may only represent a significant risk of AMD progression when co-occurring with RPD, conferring a risk even higher than that of eyes with RPD alone. This may represent a different AMD phenotype with possible genetic associations that may need further evaluation. Given the limited numbers of eyes with both CD and RPD, these findings should be interpreted as exploratory and hypothesis-generating and require confirmation in additional studies.
