# Supplementary material for: Cuticular Drusen and the Risk of Progression to Late Age-Related Macular Degeneration: A MACUSTAR Study Report
Source: Ophthalmol Sci. 2026 Aug 18;6(10):101375. doi: 10.1016/j.xops.2026.101375 (PMC13627127; doi:10.1016/j.xops.2026.101375)
Supplement: Supplementary file 1 [file mmc1.pdf]

Table S1. Baseline characteristics by country of recruitment.

	Denmark	France	Germany	Italy	Netherlands	Portugal	United Kingdom	Overall
**Age (years) [mean, sd]**	74.1 (4.7)	73.5 (7.4)	71.4 (7.2)	70.6 (7.1)	71.5 (7.4)	73.7 (6.2)	72.2 (7)	72.1 (7)
**Sex (female) [N, %]**	16 (53.3%)	45 (72.6%)	146 (69.9%)	56 (64.4%)	32 (58.2%)	45 (62.5%)	49 (70%)	389 (66.5%)
**RPEDC volume (mean, sd)**	-0.0501 (0.649)	-0.0183 (1.15)	-0.0624 (0.855)	-0.0169 (1.32)	0.183 (1.17)	-0.139 (0.638)	0.218 (0.994)	-0.00441 (0.991)
**Presence of RPD (N, %)**	9 (30.0%)	20 (32.3%)	43 (20.6%)	25 (28.7%)	22 (40.0%)	14 (19.4%)	25 (35.7%)	158 (27.0%)
**Presence of PA (N, %)**	17 (56.7%)	26 (41.9%)	105 (50.2%)	47 (54.0%)	34 (61.8%)	22 (30.6%)	36 (51.4%)	287 (49.1%)
**Presence of HRF (N, %)**	12 (40.0%)	28 (45.2%)	106 (50.7%)	42 (48.3%)	30 (54.5%)	19 (26.4%)	41 (58.6%)	278 (47.5%)

RPEDC volume shown as z-scores (standardized).

Abbreviations: SD: standard deviation, RPEDC: retinal-pigment-epithelium-drusen-complex, RPD: reticular pseudodrusen, PA: pigmentary abnormalities, HRF: hyperreflective foci.
